# Three Strategies for Describing Social Interactions of Adolescents in a Multicultural Environment—Indicators for the Quality of Life Research

**DOI:** 10.3390/ijerph18158166

**Published:** 2021-08-02

**Authors:** Alicja Szerląg, Arkadiusz Urbanek, Kamila Gandecka

**Affiliations:** Faculty of Historical and Pedagogical Sciences, University of Wrocław, 50-137 Wrocław, Poland; alicja.szerlag@uwr.edu.pl (A.S.); kamila.gandecka@uwr.edu.pl (K.G.)

**Keywords:** quality of life, adolescents, social relations, multicultural environment, integration

## Abstract

Background: The analysis has involved social interactions in a multicultural environment. The social context has been defined by the Vilnius region (Lithuania), where national, religious, and cultural differences exist across generations (multicultural community). The space of “social relationships”, as one of the modules of the WHO quality of life assessment, has been studied. An innovation of the research has been related to the analysis of the phenomenon of community of nationalities and cultures as a predictor of quality of life (QoL). The social motive of the research has been the historical continuity (for centuries) of the construction of the Vilnius cultural borderland. Here, the local community evolves from a group of many cultures to an intercultural community. Interpreting the data, therefore, requires a long perspective (a few generations) to understand the quality of relationships. We see social interactions and strategies for building them as a potential for social QoL in multicultural environments. Methods: The research has been conducted on a sample of 374 respondents, including Poles (172), Lithuanians (133), and Russians (69). A diagnostic poll has been used. The respondents were adolescents (15–16 years). The research answers the question: What variables form the interaction strategies of adolescents in a multicultural environment? The findings relate to interpreting the social interactions of adolescents within the boundaries of their living environment. The description of the social relations of adolescents provides an opportunity to implement the findings for further research on QoL. Results: An innovative outcome of the research is the analysis of 3 interaction strategies (attachment to national identification, intercultural dialogue, and multicultural community building) as a background for interpreting QoL in a multicultural environment. Their understanding is a useful knowledge for QoL researchers. The data analysis has taken into account cultural and generational (historical) sensitivities. Therefore, the team studying the data has consisted of researchers and residents of the Vilnius region. We used the interaction strategies of adolescents to describe the category of “social relationships” in nationally and culturally diverse settings.

## 1. Introduction

### 1.1. Social Interactions as a Determinant of QoL

The research intention adopted has been based on defining strategies of building social interactions as a background for QoL research. QoL is a vague concept, which is difficult to define, so researchers rely on the descriptive approach proposed by the WHO [1,2]. Six modules were taken into account in the QoL study: (a) physical health (covering issues of health, well-being, absence of pain), (b) psychological (emphasizing self-image and self-acceptance), (c) level of independence (referring to conditions of daily life, mobility, financial independence, employment), (d) social relationships (personal and group relationships, social background available to an individual), (e) environment (i.e., living environment, job satisfaction, social and medical security, leisure opportunities), (f) spirituality, religion, personal beliefs [3]. Our research focuses on the social interaction module (social relationships) in a nationally and culturally diverse environment. 

In our research, the innovation is to show the generational context of building wider, cross-cultural relationships. Therefore, the innovation involves the described relationship-building strategies as broader social trends. In our assumption, QoL, studied and described in the context of social relations, touches the socialization of generations in a given territory, the recognition of generational transmission of the value of striving to maintain a multicultural community. Of course, QoL is a subjective feeling, but we try to show the importance of the non-individual, environmental context that forms it, even if not always noticed by the adolescents. They are not always aware that the strategies they use are a phenomenon of the living environment.

The findings of the research fill a niche because QoL is usually studied as a subjective assessment, combining everyday life and metaphysics, individuality and social resources available to us [4,5]. Researchers describe QoL in the psychological dimension using psychometric tools [6,7]. However, in our opinion, the second direction is underestimated. The subjective assessment of QoL, and especially its scientific interpretation, should recognize the diversity and historical genesis of living conditions (the sociological dimension), as well as issues of socialization and upbringing into a particular environment (the pedagogical aspect). Therefore, we pose the thesis that without a thorough understanding of social interactions in their historical and pedagogical aspects, an analysis of QoL research findings is incomplete [8,9,10]. 

In the case of the research, the environment of Vilnius, Lithuania, is a generational community, where the presence of diverse national and cultural groups is formed historically [11]. Therefore, in a QoL research, the sphere of social relationships requires an understanding of the wider community climate [12,13]. Interactions and social support resources are a reference point for the subjective assessment of QoL as an individual’s relationships with his or her environment [14]. The analyzed sense of pride, job satisfaction, evaluation of one’s own competences as components of life well-being assessment [15] take on a new dimension in multicultural environments. QoL is derived from multicultural identification with social groups and individual identification in a cross-cultural context [16,17]. It is from this perspective that interactional strategies of adolescents, which broadly describe the social background for a QoL research in nationally and culturally diverse settings, will be presented. 

An important aspect of defining social interactions is socialization into living conditions and the process of learning to cooperate in a community. In this view, the concept of well-being proposed by the Rahel Dodge’s team [18] as a balance between personal resources and one’s own goals is noteworthy. Relationships at the cultural interface encourage the adoption of a synthetic model of understanding and learning (synthesis level) [19], which integrates knowledge and experience, combining the known with the unknown, therefore learning at the interface of cultural diversity leads to non-schematic understanding [20,21], creativity, ingenuity in action [22], which fits with the convention of the QoL assessment proposed by the WHO. 

### 1.2. Multicultural Settings Described by Social Interactions

Studies carried out in multicultural settings are differentiated by their location and the associated scientific problems. A different scope of analysis is found in refugee and migrant settings, where relationships at the interface of culture and religion are relatively short-lived and dominated by social problems [23,24]. Other issues arise in the environment of borderland of nations or states, where customs, traditions, and neighborly relationships intermingle. However, the presented research has been situated in the historically and spatially enduring multicultural environment of Lithuanian society, where culturally diverse communities have existed within one state for centuries. In this aspect, our commitment to building the data analysis team should be highlighted. Inhabitants and ethnographers of the Vilnius region have been its important members. The head of the research team has been exploring the issue of relations in the Vilnius region for several decades. The research is an image of the phenomenon of environmental community, but it also touches on the issue of cultural sensitivity. We are studying minorities, so the culture, tradition, and historical context of the observed data are a natural part of the methodological process [25].

Explorations in Canada, USA, Turkey, China, Brazil, Thailand [26], Indonesia [27] Malaysia [28] and Bosnia and Herzegovina [29,30] are interesting examples of the research direction we have adopted in multicultural environments. Researchers emphasize the timelessness and the implementable nature of the studies carried out in the conditions of enduring, generational communities composed of separate cultures. The issue of relationships and interactions between cultures is often included only as a background for QoL studies [31,32], and there is an assumption that they are described and understood. However, social interactions are rarely recognized as an autonomous research task. We are therefore looking for the rules that govern them, and for the strategies for creating and maintaining them. For us, multicultural interactions are an important factor that is necessary to understand in the context of the phenomenon of a given social environment. It is important for researchers before they start direct readings of QoL in environments, treated as phenomena.

It poses an oversimplification to study and define the similarities or differences of cultures without considering the motivations and meaning of the creation of a space at the interface of different cultures [33]. Especially in long-established environments, interactions serve the common purpose of creating a community, consolidating people, and harmonizing activities, through which the interaction occurs [34,35]. Such an idealistic image of community coherence is vulnerable to destabilization in the everyday realities of life. Marco Antonisch and Tatiana Matejskova rightly point out that integration can be limited only to one’s own groups and then tends towards fundamentalism and domination. Integration is not a solidarity determined only by identification with one’s own group against another [36], and such processes undoubtedly impinge on perceptions of QoL. These relations are also threatened by the actions of state authorities, which may promote one ethnic or cultural group and thus introduce unrest and conflicts regarding privilege and discrimination. It is not without significance that UNESCO, in its Universal Declaration of Human Rights, emphasizes that arguments about cultural diversity cannot be a reason for restricting the rights of any human being [37]. Integration and coherence in multicultural environments can have good and bad sides, so it is important to describe it as a process and not as a one-off snapshot of reality. From our research perspective, the interpretation of QoL always requires a reference to these realities. 

Our research has a pedagogical dimension, so we treat interaction strategies in ethnically, nationally, racially or religiously diverse communities as a contribution to the understanding of conditions of socialization in families, of upbringing and multicultural education. The term community is important here, because we relate it to those environments that are formed generationally; for us, they are the studied phenomenon. An example is the ethnographic study of the Banuroja community in Indonesia, which has been an example of such a community for generations. The name of the community itself is an acronym describing the origins of the population of Bali, Nusa Tenggara, Gorontalo, Java; has 1073 residents, combining Muslims, Indians, Protestants, and Catholics. The authors of the study point out that it is a unique community; it is an extraordinary model of building relationships in the community. However, important lessons emerge from this ethnographic research, indicating generationally developed rules for maintaining relationships. The conditions for their conflict-free existence are: (a) respect for different values with the right to accentuate, express, manifest them, (b) inclusion of religious values in everyday activities to emphasize the group identification of its members, (c) maintenance of personal relations between ethnic groups, (d) use of different languages in everyday life and creation of a system of intergroup communication, (e) activity of people to get to know others, real involvement in maintaining mutual relations, (f) openness to co-participation in different groups, a certain degree of cultural and religious syncretism, (g) the role of social and religious activists, who encourage the inhabitants to care about diversity as a community value and take on the burden of mediation in conflict situations [27]. The research draws general conclusions for understanding and building interactions, which include: (a) an activity in celebrating diversity, (b) a commitment to learning about each other. In addition, Lawrence A. Blum points to: (a) a desire to respect differences, (b) a respect for and desire to actively learn about each other, (c) a sense of happiness to live in a diverse community [38]. 

Interpreting interactions in relation to longer, generational time frames is a space for overcoming stereotypes, prejudices and conflicts, but also a challenge. Interaction requires guaranteeing the equal status [39] of participants, intensifying direct friendship contacts, which, according to studies, contribute most to mutual cognition [40]; it also requires task interdependence that motivates to achieve common goals. Therefore, when describing the potential of social relationships, it is important to recognize their importance for activation towards common goals [41,42]. One of the features of relationships in multicultural environments is the relatedness between cultures that Erich Fromm [43] points out, and its image is religious or cultural syncretism. It is accentuated in the study of the Banuroja community, where the residents read books on Islam and Christianity regardless of their religion, because they see this diversity as a source of knowledge. However, syncretism is also evident in Bosnian villages, uniting Catholics, Orthodox believers, and Muslims for centuries [29,30]. This applies, for example, when Muslims place their own symbols on the graves of Christian friends and Christians burn candles for the souls of dead Muslims, even though candles are not used in Islam as a form of remembrance of the dead. Perceptions of QoL are formed by the need to maintain one’s own ethnic identifications in the living environment [44,45,46], but also by attitudes and a real commitment to sustaining community, a willingness to trust one another and to manifest respect for otherness [47]. These are two important trends, the importance of which our research has confirmed.

## 2. Research Method

The object of the research is an analysis of social interactions of young people in a multicultural space. The phenomenon studied is an environment in which the diversity of cultures, religions, and nationalities has existed for centuries and is generational in nature. Therefore, even though we study young people, the analysis of the data needs to take into account their generational context. We do not explore QoL directly, but the space of “social relationships”, as one of the modules of the WHO quality of life assessment. We set two research objectives: (1) identifying strategies for generational building of interactions in a nationally and culturally diverse environment, (2) analyzing the role of interactions as a social background for the QoL study. The research is intended to answer the question: What variables form the interaction strategies of adolescents in a multicultural environment? 

The tool has been developed on the basis of previous research results in the Vilnius region. For the past 25 years, the team has been conducting research to state the factors that determine the everyday activity of its inhabitants at crossroads of cultures. The exposure for the assessment of QoL and social relations has been the Polish national minority. The sought factors describing social relations in these intercultural conditions have been aimed at constructing meaningfully broader dimensions of socio-cultural (self-)identification of young Poles, among Lithuanians and Russians, in one territory [48]. The questionnaire constructed for the purpose of the research has been in line with the reality of the multicultural environment of the Vilnius region. Representatives of the local community from various nationalities and ethnographers of the Vilnius region have also been involved in its construction, so that the tool is adapted to the reality of the exploration site. 

### 2.1. Participants

The research sample has also been gender diverse. This is because the research involved: 193 girls (51.60%) and 181 boys (48.40%), which has translated into the following differentiation of the national groups studied: Poles: 91 girls (52.91%), 81 boys (47.09%); Lithuanians: 68 girls (51.13%), 65 boys (48.87%), and Russians: 34 girls (48.28%) and 35 boys (50.72%). They live in the territory of the city of Vilnius or in municipalities in the close vicinity of the city, which are characterized by national diversity of their inhabitants. The participation in the study has been voluntary. Prior to the conducting of the research, information meetings have been held with young people in schools, concerning the purpose of the research, as well as the people and institutions involved in the data collection and analysis procedure. A large role has been attributed to the generational significance of this research, as this is now the next generation being studied by the team. The participation in the study has been anonymous; 450 questionnaires have been distributed of which 374 questionnaires have been returned, with no opportunity to obtain information on the reasons for refusing to participate in the survey.

A diagnostic poll has been used; 374 respondents (aged 15–16) from different national groups, living in a common territory, namely Vilnius region, Lithuania, have participated in the survey. The sample consisted of: Poles (172), Lithuanians (133) and Russians (69). These national groups are dominant in the national structure of the Lithuanian state [49], and their concentration of residence is in the Vilnius region [50]. Importantly, they have daily, direct social relationships with each other in the residential environment. We have subjected the obtained empirical material to a two-factor analysis of variance, which has made it possible to determine the impact of the variables and their interaction effect in the form of a defined strategy of action of the studied individuals [50]. 

### 2.2. Materials

The theoretical basis for the tested strategy model in social relations has been the paradigm of coexistence developed by Jerzy Nikitorowicz (a Polish researcher of interculturalism and borderland) [51]. It contains potential factors creating generational interaction building in a nationally and culturally diverse environment. The model of such intercultural relations presupposes the existence of peace-oriented values in the environment, teaching of understanding, communication and tolerance, initiating the need for getting to know each other, cooperation and dialogue in order to preserve and shape peaceful solutions. 

Of central theoretical importance to us has been the broad meaning of the category of “interculturality”. We have defined it as a common space “between cultures”, being the result of processes of interpenetration and mixing of different cultures, in a specific place, with its specific historical, political, and social connotation. It also includes a shared community understanding of the ground for effective communication between people wishing to maintain their cultural diversity in an intercultural dialogue. Only within this category have factors (variables) been tested that statistically significantly describe social relationships and, consequently, are arranged in the strategies of their construction by the adolescents. The prospect of a generational continuation of this type of research is important here, as the variables considered have been present in these relationships for several decades.

The tested statistical model consisted of two leading (independent) variables, categorizing the tested sample: (a) nationality of the respondent (Lithuanians, Poles, and Russians) and (b) cultural type of the adolescent’s family of origin, derived from the James Percy Fitzpatrick’s leading theory [52,53]. The model first tested the correlations of the two leading variables by determining the importance of the adolescents’ national identification. At a further stage, the leading variables were correlated with additional variables such as: dialogue orientation, national identity, transnational identity, citizenship, patriotism, community of goals, religion, readiness to act for integration, education for integration. As a result of a factor analysis carried out, each variable has been marked in terms of frequency of occurrence (calculation of mean). The interaction strategy model has included only statistically significant (*p* < 0.05) variables. The data analysis has taken place with representatives of the studied community to avoid falsification of conclusions due to cultural difference [25]. 

On the basis of more than 25 years of research of the Vilnius region so far [48], a catalogue of statistically tested factors by which we describe social relations have been created. The following have been taken into account: nationality of the respondents, cultural type of the respondents’ family of origin, dialogue orientation, citizenship, patriotism, national identity, transnational identity, community of goals, religion, readiness to work for integration, and education for integration. 

### 2.3. Procedure

The interaction building strategy model has included only statistically significant variables (assuming *p* < 0.05 and only these are included in the text). Thus, the factors of dialogue orientation (*p* = 0.029), national identity (*p* = 0.013), and education for integration (*p* = 0.032) have been included. Not statistically significant (at *p* > 0.05) variables have been: transnational identity (*p* = 0.763), citizenship (*p* = 0.496), patriotism (*p* = 0.230), community of goals (*p* = 0.368), religion (*p* = 0.551), and readiness to work for integration (*p* = 0.870), which have not been included in the interaction strategy model. 

The statistical analysis has been a two-factor ANOVA, used to test the significance of the association of two classifying factors (independent variables), divided into multiple levels, with the values of a dependent variable. The method has allowed us to assess the independent effects of the two factors (main effects) and the effect of the combination of their values (interaction effect). The results of the analysis are presented as values of means in subgroups with 95% confidence interval (in tables and box plots). The significance of effects is determined by a *p* < 0.05. The necessary assumptions of ANOVA are normality of distribution and homogeneity of variance in subgroups defined by the levels of the classifying factors. In some subgroups, slight deviations from these requirements have been observed, which do not disqualify the results obtained.

### 2.4. ITC Recommendations

The research team has consisted of Poles, and the research site has involved the former Polish borderlands. Therefore, the participation of representatives of the local environment, explaining the conclusions in the light of their generational (historical) context, is important. The analysis of findings has been performed with the participation of the local community (Ciechanowiszki) and the Lithuanian ethnographer of the region.

The use of the questionnaire in the intercultural relations’ research has respected the recommendations of ITC 2000 and 2017, in terms of using the tool to optimize its effectiveness and the reliability of the data. Included in the scope are:

(a) Guaranteeing the professionalization of the research team, including people representing the local environment, having participated for 25 years in periodical surveys of social relations in this region,
(b) Taking responsibility for the effective use of the survey instrument, which is why so much attention has been paid to information meetings with young people prior to the conducting of the survey,
(c) Assessing the utility of the tool on the basis of previous generational research on social relations in cross-cultural settings, also involving ethnographers of the region,
(d) Analysis of the survey results involving regional and national representatives [54]).

We have made efforts to optimally implement the tool (survey questionnaire) to the conditions of the studied social environment. In this respect, the following have mainly been taken into account:

(a) The necessity to constantly adapt the tool to the social reality, taking into account that our team of researchers has been carrying out this type of analysis continuously for 25 years in the Vilnius region,
(b) The translation of the tool into the participants’ languages, including native speaker assessments of the questionnaire, in order to adapt the language to the social and cultural realities of the region,
(c) Respect for local laws on the conduct of these types of research involving young people [55].

## 3. Results

### 3.1. Indicators Defining a Multicultural Environment

The study of social interactions in multicultural conditions has been subordinated to three indicators: (a) social relations, (b) level of identification with one’s own national and cultural group, (c) place of living. The adolescents have represented three prevailing nationalities, generationally located in the Vilnius region: Lithuanians, Poles, and Russians. They live in the Vilnius region, i.e., in an environment whose attributive feature is national diversity. In the population of the Vilnius region, there is 59.4% of Lithuanians, 23% of Poles, and 10.3% of Russians, as well as 7.3% of other nationalities [49,50]. What is important here is the specificity of the social relations established between members of these national groups, which have been daily and permanent for generations. When describing mutual relationships as a background for QoL, there can be mentioned their local character, familiarness, and the sense of community in a living environment. Positive relationships between the respondents are also characterized by the absence of language barriers to mutual communication. They speak fluent Lithuanian, the national language, and Polish and Russian are often used by them in direct contacts. An indicator of these relations is also the generational national diversity in the families of the respondents (grandparents, parents, and children). As a result, it has been found that in the case of the studied:

- Lithuanian families: 63.2% of them are of homogeneous nationality and 36.8% are of diverse nationality,
- Polish families: 49.42% are nationally homogeneous, while 50.58% of them are nationally diverse,
- Russian: 23.19% are nationally homogeneous families and 76.81% are families with nationally different members.

The national differentiation of families, in a generational perspective, was determined by belonging of their members to the prevailing national groups in Lithuania (Polish, Lithuanian and Ruthenian), and less frequently to the Ukrainian and Belarusian national groups, which have also been historically present in the area.

The diverse living conditions of the adolescents create a social background for their identification with their own group and other neighboring national groups. The identification takes place precisely through their daily and historical presence in the living space, as well as through the processes of socialization and upbringing in their families. Families of the respondents are present here constantly and together, in close proximity without clear demarcation, linked by a common history and common destiny, as well as socio-cultural similarity [56]. The identification of young people with the community has been longitudinal (relationships within a generation) and transversal (relationships between generations). In all generations, national differentiation and existence in this space of differentiation has been present. Therefore, their national identification is more formed by the feeling of belonging to a particular national group than by the formal question of Lithuanian citizenship, which is common to all respondents. For 97.7% of the Lithuanians surveyed, it has been the Lithuanian nationality, among 94.8% of the Poles it has been the Polish nationality, and for 72.5% of the Russians, it has been the Russian nationality. In the case of the latter group, the respondents, more often than others, also indicated belonging to the Lithuanian (15.9%), Polish (8.8%) and Belarusian and Ukrainian (1.4% each) national groups. 

We therefore put forward the thesis that an important descriptive indicator for QoL is the multicultural environment, as a place to live, whose specificity is determined by:

- History formed as a result of long-term social and cultural processes,
- Significant cultural intermingling (including similarities) specific to cultural groups living in a common area,
- Absence of sharp divisions and borders between culturally diverse groups,
- Evolving from a situation of cultural domination to acceptance of the multiplicity of cultures and their equality, with rather vertical intercultural and identity relations, and the important, though not constitutive for social divisions, role of religious differentiation [57].

They form the cultural context in which social relations are established, cultural identifications are made and living conditions persist or change.

### 3.2. Cultural Type of Families in Nationally Diverse Environments

In defining the multicultural environment, the leading theory proposed by James Percy Fitzpatrick has been adopted, which assumes that the observation (study) of the process of blending families into a given culture helps to explain visible manifestations of diversity observed in their lives. Different national or regional traditions are perceived in the life of the family, thus the image of the family evolves over time. On this basis, J. P. Fitzpatrick and M. Plopa identified basic patterns of family adjustment to the process of cultural assimilation [52,53]. They has become the reference for the conceptualization of cultural types of families carried out for the purposes of the research (situating them as one of the correlated variables). Already the use of a cultural typology of families in the research allows for a fuller understanding of the real background constituted by social interactions in the living environment for the adolescents’ QoL. They have attributed themselves to the following characteristics when assessing their family. Thus, the cultural typology of their families has become the variable classifying the respondents. In the light of the leading theory, five cultural types of family can be used [52,53], but as only three of them occurred in the research, we limit the scope of characteristics. As a result, adolescents have described their families as the following cultural family types [51]; no one has assigned themselves to a culturally appropriative or deconstructed family type:

(1) Culturally open type (in the family, cultural, national and religious differences of family members are recognized, different traditions, customs, and values are respected, the family strives to develop common cultural content, which influences everyday life and upbringing of children. The cultural diversity of family members is not a cause of conflict within the family and is expressed in the positive bonds established between them);
(2) The multicultural type (in the family, there is a real national diversity of its members and acceptance of their cultural differences, contents of different cultures permeate each other creating a new cultural quality in the space of family life, multicultural orientation is present in important spheres of the family’s life, also in the upbringing of children, cultural differences do not trigger conflicts, and bonds within the family are positive);
(3) The culturally closed type (only one culture dominates in the family, at the same time, there are strong ties within the family, all family members submit to it without exception, upbringing of children takes place in accordance with the dominant culture in the family, cultural differences are not the cause of conflicts in the family).

The inclusion of a typology of cultural living of families stems precisely from our guiding thesis that QoL requires consideration of the pedagogical aspect of human socialization and upbringing into a particular living environment. Social interactions are the backdrop to the QoL research, especially in settings where children are socialized over generations to specific conditions. Based on the stratification function of socialization, we hypothesize that the studied adolescents naturally reproduce certain intergroup relations and communication patterns. They take them over from their grandparents and parents, which forms their personal interaction building strategies. Consequently, without the understanding of social relationships, it is impossible to describe QoL as their derivative.

We therefore analyze the context of adolescents’ family living conditions, which we differentiated in our study with the “cultural family type” variable. The culturally open type has prevailed in every surveyed national group. It has been indicated by Lithuanian youth: 57.89%, Polish youth: 46.51%, and Russian youth: 43.48%. It is significant that the multicultural type has been as popular as the culturally open one, with the highest number of indications in Russians (43.48%), followed by Poles (31.98%), and as third in the group of Lithuanians (20.30%). On the other hand, the occurrence of the culturally closed type in the respondents’ families has been basically associated with the national homogeneity of the families: 21.81% of Lithuanians, 21.51% of Poles, and 13.04% of Russians have indicated this type of cultural family life.

Thus, an important environmental feature in a QoL research is the consideration of family composition as diverse or homogeneous nationally, but also culturally and religiously. The existence in a given family type itself has resulted in a specific self-assessment, as the young people assigned themselves to a given cultural family type, matching their assessment to the presented catalogue of choices. 

### 3.3. Adolescents’ National Identification Attachment Strategy in a Multicultural Environment

**Figure 1:**
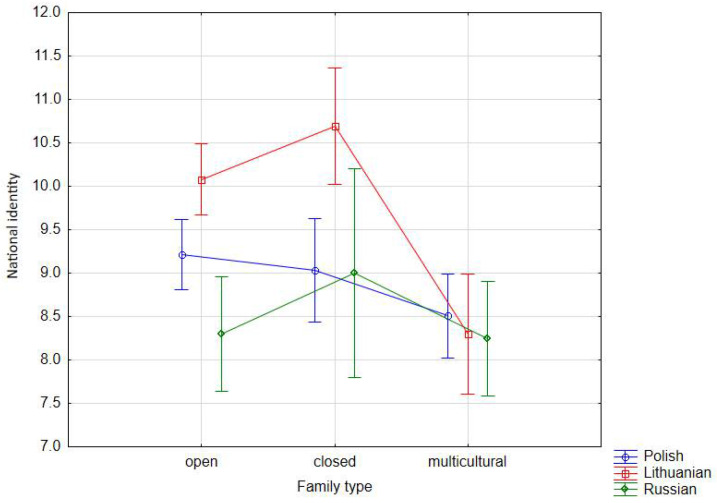
Interaction effect of nationality and family type on national identity—averages across nine combinations of nationality and family type (vertical bars denote 0.95 confidence intervals).

We have observed the first interaction strategy by correlating the variables of nationality and family cultural type. The strategy has focused on the issue of the respondents’ national identification and proved to be a clear strategy as its relevance has been evidenced by already conducted studies on multicultural communities [44,45,46,47]. In the case of Lithuanians (Table 1, Figure 1), their national identification was primarily determined by living in the culturally closed family type ($\bar{x}$ = 10.69), followed by the culturally open family type ($\bar{x}$ = 10.08) and, to a lesser extent, the multicultural family type ($\bar{x}$ = 8.30). 

Similarly, such relationship, although with a lower values of means, is found in Russian respondents, where their national identification is influenced by living in a family of the culturally closed type, and therefore, with one culture—Russian ($\bar{x}$ = 9.00) and of the culturally open type ($\bar{x}$ = 8.30). Interestingly, the culturally open family type has not reduced the sense of identification, a determinant of QoL that was confirmed in previous studies. Despite the diversity of the family, a sense of identification and belonging has emerged as an important part in the lives of the respondents. Equally important, from the educational perspective, is that the sense of identification did not wane with the cultural openness of the family. Conversely, the unifying influences of power or politics become redundant, as increasing the hermeticity of families does not radically increase the sense of belonging. Thus, the national, cultural, and customary unification of families in multicultural environments does not provide the authorities with a guarantee that people will feel more attached to their nationality than they already do in diverse families. Although the perception of Russian youth as participants in multicultural families has decreased the strength of national identification with Russian culture ($\bar{x}$ = 8.25), still, this interaction has remained statistically significant, i.e., the sense of national identification persists. In the group of Polish adolescents, the strength of national identification is greater in the case of members of families of the culturally open type ($\bar{x}$ = 9.22) and of the culturally closed type ($\bar{x}$ = 9.03) (Table 1). It is similar to Russian adolescents—the multicultural family type reduces the strength of national identification, but it is still statistically significant. 

### 3.4. Social Interactions’ Strategy Based on Intercultural Dialogue 

**Figure 2:**
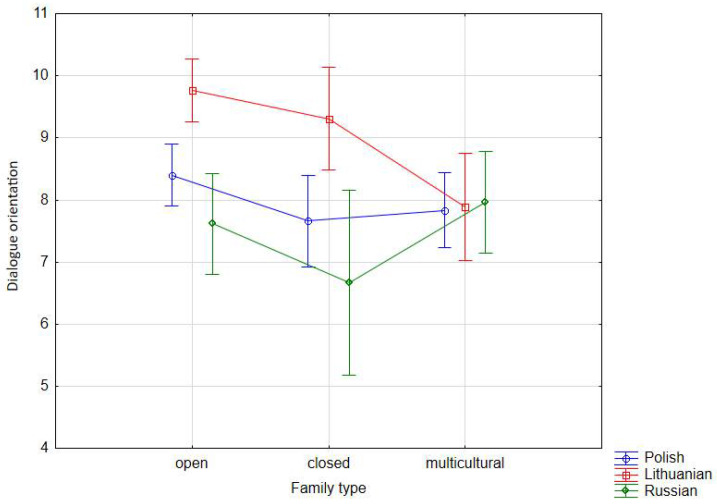
Interaction effect of nationality and family type on dialogue orientation—averages across nine combinations of nationality and family type (vertical bars denote 0.95 confidence intervals).

When correlating the leading variables (nationality, family cultural type) with the additional variables, the dialogue orientation variable has become the most prominent (the others have been statistically insignificant). A strategy of building interactions by adolescents, based on a dialogue between nationally and culturally diverse groups, has therefore emerged. We emphasize that, statistically, the adolescents’ orientation towards dialogue in a diverse environment still correlated strongly with the cultural type of the family (mean considered only at the level of statistical significance at *p* = 0.027 to *p* = 0.029). Therefore, the conditions of the family of origin, and thus the socialization of generations into the community, potentially plays an important role for describing QoL. The formation of dialogue-based interaction strategies was strongest in the culturally open ($\bar{x}$ = 8.59) and multicultural ($\bar{x}$ = 7.90) families. Although somewhat surprisingly, families of the culturally closed type are only slightly less likely to form this orientation ($\bar{x}$ = 7.88). The general trend allows to put forward a thesis that the dialogue strategy is not only part of the family culture, but rather an environmental requirement. Generational relationships enforce its existence as an important social resource, an important potential for activity in the local environment to which family systems are subordinated. This is also shown by the data generated for each national group. For Lithuanian families (Table 2), the dialogue orientation strategy is the domain of the culturally open families ($\bar{x}$ = 9.77) and slightly less of the culturally closed ($\bar{x}$ = 9.31) families. Surprisingly, the lowest occurrence of dialogue orientation is in families of the multicultural type ($\bar{x}$ = 7.89). In Polish adolescents, the dialogue interaction strategy is present in the culturally open families ($\bar{x}$ = 8.40), followed by the multicultural families ($\bar{x}$ = 7.84), and of the culturally closed type ($\bar{x}$ = 7.66), which could be modeled as an effect of the defined socialization. In the group of Russian adolescents, the dialogue strategy in building social interactions was associated with socialization in multicultural ($\bar{x}$ = 7.97) and culturally open ($\bar{x}$ = 7.61) families (Table 2, Figure 2).

The dialogue strategy in diverse environments becomes the spectrum of reference for the QoL assessment as it is a resource of mutual relationships. Particularly for further QoL research, there is a catalogue of factors forming the strategy of intercultural dialogue (factor analysis with the method of principal components with varimax rotation has been applied for the assessment of thirteen factors whose loadings has been above 0.40 or above −0.40). Thus, when conducting a research on QoL in a diverse environment, the background of social interaction is determined by factors such as: interculturality—interpenetration of different values, behaviors, traditions, and customs creating common platforms for interaction and social communication (0.82), cooperation despite cultural differences (0.81), openness to other cultures (0.79), transnational understanding (0.75), cultural community (0.74) and respect for the common cultural heritage (0.56). In addition, the dialogue strategy draws on certain values and tasks present in a given local environment, including: religious values (0.76), activities for the local community (0.68), building the common culture (0.57), respect for the common cultural heritage (0.56), and familiarness, i.e., the recognition of the multicultural specificity of the living environment as one’s own, thanks to which the process of socialization through culturally diverse social relations takes place (0.54). On one hand, they should be regarded as important determinants of social relations established by members of different national groups in a common living environment. On the other hand, the analyzed values create an intercultural space of the adolescents’ existence and their QoL in a multicultural environment.

### 3.5. Strategy of Engaging in Community Building in a Multicultural Environment by Adolescents

The research we represent has primarily a pedagogical dimension, so the third strategy touches on the issue of community-based multicultural education. It emerged as the sum of the above relational strategies. On the basis of the attachment to national identity and orientation towards neighborly dialogue, it was possible to form community-building strategies across generations. We see it as a socializing effect of generational living in the community and as a future perspective of multicultural education.

The strategy of activity and commitment to community building has two dimensions. One is to strengthen the sense of civic bonding, despite strong identification with one’s own national group. The adolescents attach great importance to what is common, i.e., patriotism towards Lithuania (0.90), respect for the Lithuanian homeland (0.88), and active citizenship (0.68). Thus, regardless of the respondents’ affiliation to certain cultural groups, identification with the Lithuanian homeland locates their social relations on the civic level. 

The second dimension of this interaction strategy is the everyday nature of relationships between national groups and the interpenetration of their cultures. Generational community of the environment becomes a value for adolescents, who emphasize: the importance of direct and positive social relations, establishment of relations between nationally different members of the community, and socialization of generations into the conditions of a multicultural place of life. The specificity of the living environment community is also a derivative of: the axiological similarity, localness, and familiarness of interactions, present in everyday life. The third dimension, most interesting for a study of QoL and its understanding by adolescents, is the link between the community strategy and education for the integration of a multicultural environment. 

Taking into account the national identification of the respondents, the need of education for integration has been indicated primarily by Lithuanians ($\bar{x}$ = 27.88) and Poles ($\bar{x}$ = 27.56), and to a lesser, although significant, extent by Russians ($\bar{x}$ = 24.49). The above can be seen from the perspective of national dominance in Lithuanian society or remaining in the position of a national minority with the conviction, however, of the historically sanctioned right to socio-cultural equality. Each of these perspectives aims to build community at the crossroads of cultures. Hence we attribute great importance to education for integration. The family of origin, and especially its cultural functioning, plays a significant role in such educational influences. As the results of the study show, it is, above all, the families of the multicultural ($\bar{x}$ = 27.87) and culturally open ($\bar{x}$ = 27.61) types that are the ones in which importance is attributed to upbringing for integration. To a lesser, but statistically significant extent, the importance of this upbringing can also be referred to families of the culturally closed type ($\bar{x}$ = 25.44). It appears that the very existence of adolescents and their families in a culturally diverse environment stimulates actions aimed at preparing the young generation to function in that environment. Thus, two factors: the power of identification and the drive to sustain community coalesce in the picture of social relations. Diversity becomes an authentic value and a social potential. Of course, such situation concerns precisely the character of generationally (historically) differentiated environments. This also supports the central thesis of the study that without a thorough understanding of the social background of a multicultural environment, it is impossible to understand subjective assessments of QoL.

**Figure 3:**
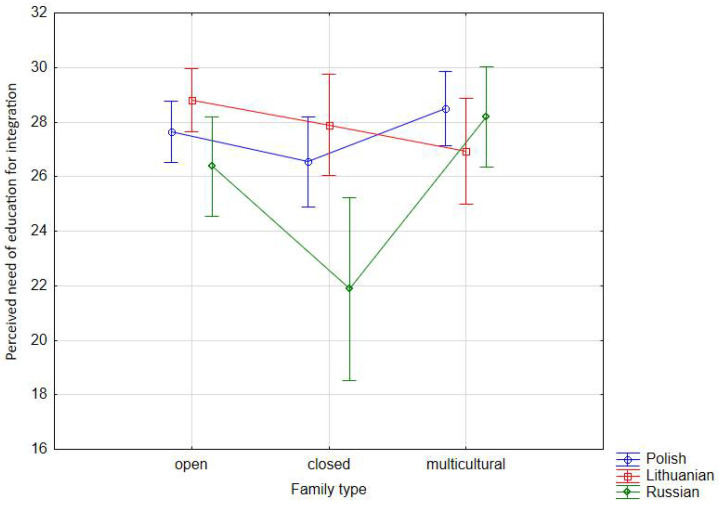
Interaction effect of nationality and family type on the perceived need of education for integration—averages across nine combinations of nationality and family type (vertical bars denote 0.95 confidence intervals.

In Polish and Russian families, these dominations are similar. Families of the multicultural and culturally open type attribute the highest importance to the need of upbringing for integration. Surprisingly, this direction of upbringing is also (although actually at a lower level) in families of the culturally closed type, maintaining a statistically significant trend. On the other hand, in the case of Lithuanian families, ascribing importance to the need of upbringing to the community occurs primarily in families of the culturally open type, less so in the culturally closed type and least in the multicultural type. Taking into account the statistical means, the greatest importance to upbringing for social community is attached by families of the culturally open type in the case of Lithuanian families of respondents ($\bar{x}$ = 28.81), of the multicultural type in the case of Polish families ($\bar{x}$ = 28.49), and of the multicultural type in Russian families ($\bar{x}$ = 28.20) (Table 3, Figure 3).

An important conclusion for the study of QoL social resources is that regardless of the type of family culture, the adolescents have believed that they are prepared by their parents to continue living in the community. Regardless of political turmoil, aspirations for greater or lesser unification, the generationally-formed community is internally immune to these pressures. On the other hand, the community nature of the living environment has proven to be of value not only for young people as their grandparents and parents continue to form communal attitudes through proper socialization and upbringing of their children, replicating them in every generation.

In a broader sociological and pedagogical context, community strategy in a multicultural environment is the result of an intertwining of historical experiences, attitudes, and perceptions of living space. It is constituted by: (a) generational experience of cultural differences in the family and living from generation to generation in a multicultural environment, (b) commonality of fates and existence of nationally different members of the local community, (c) entering into direct social relations with members of different cultural groups in the local environment, (d) sense of community at the meeting point of cultures, determined by the locality and familiarness of this meeting point, (e) citizenship of adults and adolescents oriented towards the common homeland and state.

## 4. Discussion

The discussion on the research results draws attention to their predictive dimension, which is part of a broader perspective on the problem of multicultural communities, interaction strategies represented in them and, consequently, QoL of the members of these communities. On one hand, by planning research on QoL in a multicultural environment, we gain concrete indicators that can be used to interpret the living environment. The three adolescent strategies define social interactions, implementable as indicators of variables. On the other hand, when analyzing QoL, it is important to take into account issues of socialization and upbringing, generationally forming a specific understanding, construction and use of a multicultural community. Consequently, multicultural environments do not reconcile to unified research approaches. The solution is based on precisely defined interactions, without which a QoL survey does not provide a full understanding of the respondents’ evaluation perspective. 

In the 1980s, Manuel Castells took up a similar theme of analyzing relationships, showing the importance of new orientations of societies in the clash with projected crises. He categorized the increasing urbanization, elimination of a sense of community in favor of anonymity and issues of ecological risks to regions as threats [58]. The answer to the crisis was to be a new orientation of the societies, which appreciated the traditions, the history of the region, and above all, the process of building continuity between the society and its living space. In our research, we want to show precisely this aspect of social relations, defined in the wider context of region, tradition, history, and generation. An important point is the third strategy we have described, namely the pursuit of community. We recognize the need of adolescents to go beyond their national, ethnic group, and on the other hand, the value of the Vilnius society is to incorporate care for the community into the socialization of generations. We are aware that the studied area is a phenomenon, therefore our analyses can be implemented in QoL research in traditional, intercultural environments. Thus, contemporary responses to the crisis of ecology or the instrumentalization of culture as a threat, according to Manuel Castells, require strength and a union of collectivities. Here, the intercultural community is a phenomenon, a value beyond everyday conflicts and conflicting aspirations. In our view, the described strategies are an example of the different orientations of the local society towards an intercultural community. 

In our research, the innovation is to show the generational context of building wider, cross-cultural relationships. It is not just about instrumental, task-based relationships, but about historically grounded messages and relationship-building strategies. We understand strategies as broader social trends, along with their generational, historical, and pedagogical contexts. We try to show trends that override an individual’s assessment of the frequency of contact and the breadth of the group. When discussing research results, we see them as a resource, a value for the future. In this respect, we try to show a continuation of the understanding of social relationships and QoL as the adolescents’ orientations to everyday life and the future [59]. Dennis Raphael sees links between the initiation of future-oriented activity with the sense of QoL. However, he also recognizes it as an important determinant of the adolescents’ health and mental condition. Living in an intercultural community and the efforts to sustain it (which have been present for generations) are, in our opinion, such an activity towards the future. The results of Raphael’s research show that the QoL assessment of adolescents includes two important indicators: (a) the sense of social belonging (on a macro scale) and (b) the sense of belonging to the living environment (on a micro scale). In our research, we show that it is impossible to stop at general social belonging (national, Lithuanian—strategy 1), because next to them, there appeared a generationally fixed strategy of building relations in the immediate space of life (strategy 2). They exist side by side, they do not collide or exclude each other, but they form a task, because the community is sought and cared for.

An important area of crisis in contemporary societies is also the superficiality of social policies for QoL. Multiple social policy tools, monetary support, and sociocultural effects such as accessibility to support and personal development cannot always be equated with understanding of a personal sense of QoL [58]. In our view, it is about the emotional resources of the living environment community, which do not lend themselves to easy measurement like social tools. In addition, the state policy is important, which, in culturally diverse societies, can oscillate between repressiveness and integration, thus using social support tools for political ends. Therefore, when studying the adolescents’ QoL, it is worth taking into account the strategies that they use in relationships, as these are a different space from formal resources. The young generation of Poles, Lithuanians, and Russians, living in the multicultural society of Lithuania, are active in a public space, in which a social distance is present (with varying degrees of intensity), manifested by Lithuanians especially towards the Polish national minority. It is conditioned, above all, by the historical background, contemporary internal politics of the Lithuanian state, and Lithuanian nationalism. Many of the rights of national minorities in Lithuania are not respected (e.g., learning their own language, bilingual signs of municipalities, spelling of names and surnames, the right to religious worship, or the right to participate in public life). The predominant causes of conflict, on the other hand, are primarily political in origin, i.e., they are imposed inspirations, which are external to local communities. This is why our research builds a different perspective of QoL; it is understood locally, experienced in micro-communities. Despite the radicalizing policy of the Lithuanian state towards national minorities, nationally diverse local communities are active in direct and positive relations. People’s QoL is derived from their generational (historical and pedagogical) actions to neutralize external political influences and to develop an openness to cultural differences and intercultural understanding in their everyday practices. This is all the more so because cultural difference in the families of the respondents is generationally entrenched. Despite the everyday difficulties in the adolescents’ relationships, we assume, based on previous research, that the multicultural local environment is community oriented. QoL thus becomes a derivative of social interactions described in interculturality understood according to the description we have adopted. 

## 5. Conclusions

The obtained research results confirmed the thesis that understanding QoL requires a multi-level understanding of the respondents’ social relationships. Indeed, QoL can be seen as a balance between personal resources and opportunities to achieve individual goals [18], taking into account a broad understanding of these resources. The results of the study have situated the adolescents’ personal social resources (i.e., relationships, interactions, and living environment) on several levels, confirming the findings of previous research. Their interaction strategies were formed on the basis of direct relationships, dominated by friendship and task contacts. Conclusions that social relationships are both individual resources (e.g., the strategy of national identification and its importance for adolescents) and group resources, which is important for a community (e.g., the strategy of community building through cooperation between diverse groups), have been confirmed. 

In the discussion, the diverse nature of multicultural environments is an important issue, as the feature of intercultural relatedness (E. Fromm) is particularly strongly accentuated in this case [43]. It was present in the dialogue strategy and in the multicultural community building strategy. This nature of the living environment therefore links two aspects of a QoL research: interpersonal relationships and the generationally-formed living environment. Adolescents identifying themselves with Polish culture and nationality have particularly strongly shown cultural affinities (Table 1). This is because in the family environments of the respondents, Polish and Lithuanian culture coexist, hence they refer to themselves in a dual way, as Polish Lithuanians. An important role is played here by the heritage of Polish ancestors who have lived in the Vilnius region from generation to generation, the place of birth, and the common fate and culture, which together cause national identification to be related to two homelands—Polish and Lithuanian states. The national identification strategy, which draws on families of origin, is important in interpreting the QoL findings, because our results show family and generational (historical) sources determining the subjective assessment of one’s own place in the local community.

Another outcome of the research is the interpretation of relational strategies in the context of their everyday life. On one hand, the literature accentuates the aspirations of a community to celebrate diversity [27], but such direction has not been borne out in the results of our study. The second aspect has appeared to be important, i.e., the high involvement of members in getting to know each other, which has definitely been present in the described strategies. The adolescents’ social activities have concentrated on joint actions. What is important, however, is the nature of this cognition, which has taken on a “just like that” dimension, being daily and obvious. Its origins lie precisely in the role of a component of the generations’ socialization and upbringing for integration in a community. The realization of the three strategies, which have been derived from the statistical data, in a multicultural environment, has been determined by: (a) generational living in a nationally diverse environment, generating intercultural social relations; (b) lack of communication barriers as a result of members of national groups speaking the state language and languages specific to these groups, which are historically established and accepted; (c) generational national diversity of family members, originating from national groups historically living in the common area; (d) openness to cultural differences and their acceptance in the living environment, fostering the establishment of intercultural social relations; (e) open and multicultural character of the cultural family types, orienting socialization towards cognition, understanding of differences, and cooperation in a multicultural living environment; (f) a sense of community in a multicultural living environment, determined by locality, familiarness and interculturality, and (g) a generationally established tendency to consolidate and develop community, through education for integration, at the meeting point of cultures. The above premises indicate the background of the interpretation of QoL in a multicultural environment, taking into account the specificity of interpersonal relations.

A continuation of the discussion on the role of socialization and upbringing is the community’s efforts to maximize attitudes of dialogue and cooperation. Of course, studies to date accentuate opportunities and threats for a community that may struggle with privileging of a particular group, lack of equality in respecting difference, and political pressures for unification. However, the social effect of strategies based on dialogue and community is the potential for resistance to external destabilizing pressures. Therefore, an interesting continuation of the research on adolescents’ QoL could be precisely to consider the importance of the cultural types of family that they perceive. The source data (Table 1) report on specific choices and adolescents’ perceptions of their own family as open or multicultural. However, a perspective for discussion is a further exploration of the way in which perceptions of the family type interact with the social quality of their lives. Does the family type affect perceptions of the social resources available to them? What is a determinant of QoL? The data indicate that in most of the respondents’ families, there is an acceptance of the cultural diversity of its members, which results in the development of common areas for the cultural functioning of the family. National difference is therefore not a conflict-triggering factor, and common cultural content (traditions, customs, and values) is conducive to maintaining positive relations and strengthening intra-family ties. Relating the research results to the analysis of the literature, it is recognized that the openness and multiculturalism of families is becoming a new social resource, influencing wider cooperative and communicative opportunities. This cannot be overlooked in a QoL research as it explains, to a large extent, the judgements, choices, and taken actions.

## Figures and Tables

**Table 1:** Respondents’ nationality and cultural family type vs. national identity—an analysis of variance.

Subsets Separated by Values of Independent Variables		Statistics of the Dependent Variable in the Selected Subsets of the Sample				
Nationality	Family Type	Mean	Statistical Error	+95% CI	−95% CI	N
Lithuanian		9.69	0.18	9.34	10.04	133
Polish		8.92	0.15	8.63	9.21	172
Russian		8.52	0.26	8.01	9.02	69
Analysis of variance for the main effect		F(2, 365) = 8.81, *p* = 0.000 (significant)				
	closed	9.57	0.25	9.08	10.07	75
	open	9.20	0.15	8.91	9.49	187
	multicultural	8.35	0.18	7.99	8.71	112
Analysis of variance for the main effect		F(2, 365) = 9.84, *p* = 0.000 (significant)				
Lithuanian	closed	10.69	0.34	10.02	11.36	29
Lithuanian	open	10.08	0.21	9.67	10.49	77
Lithuanian	multicultural	8.30	0.35	7.60	8.99	27
Polish	closed	9.03	0.30	8.44	9.62	37
Polish	open	9.22	0.20	8.81	9.62	80
Polish	multicultural	8.51	0.25	8.02	8.99	55
Russian	closed	9.00	0.61	7.80	10.20	9
Russian	open	8.30	0.33	7.64	8.96	30
Russian	multicultural	8.25	0.33	7.59	8.90	30
Analysis of variance for the interaction effect		F(4, 365) = 3.20, *p* = 0.013 (significant)				

**Table 2:** Respondents’ nationality and cultural family type vs. dialogue orientation—an analysis of variance.

Subsets Separated by Values of Independent Variables		Statistics of the Dependent Variable in the Selected Subsets of the Sample				
Nationality	Family Type	Mean	Statistical Error	+95% CI	−95% CI	N
Lithuanian		8.99	0.22	8.56	9.42	133
Polish		7.97	0.18	7.61	8.32	172
Russian		7.42	0.32	6.79	8.04	69
Analysis of variance for the main effect		F(2, 365) = 10.25, *p* = 0.000 (significant)				
	closed	7.88	0.31	7.26	8.50	75
	open	8.59	0.18	8.23	8.95	187
	multicultural	7.90	0.23	7.45	8.34	112
Analysis of variance for the main effect		F(2, 365) = 3.67, *p* = 0.027 (significant)				
Lithuanian	closed	9.31	0.42	8.48	10.14	29
Lithuanian	open	9.77	0.26	9.26	10.28	77
Lithuanian	multicultural	7.89	0.44	7.03	8.75	27
Polish	closed	7.66	0.37	6.93	8.39	37
Polish	open	8.40	0.25	7.90	8.90	80
Polish	multicultural	7.84	0.31	7.23	8.44	55
Russian	closed	6.67	0.76	5.18	8.16	9
Russian	open	7.61	0.41	6.80	8.43	30
Russian	multicultural	7.97	0.41	7.15	8.78	30
Analysis of variance for the interaction effect		F(4, 365) = 2.73, *p* = 0.029 (significant)				

**Table 3:** Respondent nationality and cultural family type vs. perceived need of education for integration—an analysis of variance.

Subsets Separated by Values of Independent Variables		Statistics of the Dependent Variable in the Selected Subsets of the Sample				
Nationality	Family Type	Mean	Statistical Error	+95% CI	−95% CI	N
Lithuanian		27.88	0.50	26.90	28.85	133
Polish		27.56	0.41	26.76	28.37	172
Russian		25.49	0.72	24.07	26.90	69
Analysis of variance for the main effect		F(2, 365) = 4.06, *p* = 0.018 (significant)				
	closed	25.44	0.71	24.05	26.84	75
	open	27.61	0.41	26.80	28.42	187
	multicultural	27.87	0.51	26.87	28.87	112
Analysis of variance for the main effect		F(2, 365) = 4.35, *p* = 0.014 (significant)				
Lithuanian	closed	27.90	0.95	26.03	29.76	29
Lithuanian	open	28.81	0.58	27.66	29.95	77
Lithuanian	multicultural	26.93	0.98	24.99	28.86	27
Polish	closed	26.54	0.84	24.89	28.20	37
Polish	open	27.65	0.57	26.53	28.78	80
Polish	multicultural	28.49	0.69	27.13	29.85	55
Russian	closed	21.89	1.70	18.54	25.24	9
Russian	open	26.37	0.93	24.53	28.21	30
Russian	multicultural	28.20	0.93	26.36	30.04	30
Analysis of variance for the interaction effect		F(4, 365) = 2.68, *p* = 0.032 (significant)				

## Data Availability

The data presented in this study are available on request from the corresponding author. The data are not publicly available due to copyright—the publication is not open access.
